# Allergic bronchopulmonary Aspergillosis in children

**DOI:** 10.3906/sag-2104-227

**Published:** 2021-10-21

**Authors:** Özge ATAY, Suna ASİLSOY, Gizem ATAKUL, Serdar AL, Özge KANGALLI BOYACIOĞLU, Nevin UZUNER, Özkan KARAMAN

**Affiliations:** 1 Department of Pediatric Immunology and Allergy, Faculty of Medicine, Dokuz Eylül University, İzmir Turkey

**Keywords:** Allergic bronchopulmonary aspergillosis, asthma, cystic fibrosis, child

## Abstract

**Background/aim:**

Allergic bronchopulmonary aspergillus (ABPA) is a lung disease caused by hypersensitivity from Aspergillus fumigatus. Diagnostic criteria, staging systems and treatment methods for ABPA disease have been reported in studies evaluating populations, the majority of which are adult patients. Our study aimed to discuss the use of ABPA diagnostic criteria in children, the success of other alternative regimens to oral corticosteroids in the treatment of ABPA, and the changes that occur during treatment, in the light of the literature.

**Materials and methods:**

Between January 2017 and 2020, patients diagnosed with ABPA at the Dokuz Eylül University Child Allergy and Immunology clinic were identified; demographic characteristics, clinical and laboratory findings, diagnostic scores and stages, and treatment protocols were analyzed retrospectively.

**Results:**

The mean age of patients diagnosed with ABPA was 14.33 ± 1.96. At the time of ABPA diagnosis, the median total IgE level was 1033 IU/mL (1004**–**6129), and the median AF specific IgE was 10.64 (2.59**–**49.70) kU/L. Bronchiectasis was detected in HRCT of 5 cases. We detected significant improvement in spirometric analysis with omalizumab treatment in our patient with steroid-related complications.

**Conclusion:**

Today, although risk factors have been investigated for ABPA, it has not been revealed clearly. Both diagnostic criteria and treatment regimens have been described in research studies, mostly adults. In pediatric patients; clarification of diagnosis and treatment algorithms is necessary to prevent irreversible lung tissue damage and possible drug side effects.

## 1. Introduction

Allergic bronchopulmonary aspergillosis (ABPA) is a lung disease caused by hypersensitivity to Aspergillus fumigatus (AF), especially in asthma and cystic fibrosis patients [1,2]. Its prevalence varies between 2%–15% [3]. The diagnostic criteria proposed by Rosenberg–Patterson in 1977 were edited by Greenberger in 2007 and Agarwal et al. in 2016 [4–6]. In the case of fungal sensitivity accompanying severe asthma patients, the detection of total IgE level <1000 IU/mL suggests the diagnosis of severe asthma with fungal sensitization (SAFS) [7]. For the treatment of ABPA, corticosteroids, antifungal treatments, and anti-IgE, monoclonal antibodies are used [1]. We aimed to discuss ABPA diagnostic criteria, clinical and laboratory findings, and the success of different treatment regimens in the light of the literature on our children with ABPA diagnosis.

## 2. Materials and methods

Between January 2017 and 2020, patients diagnosed with ABPA at the Dokuz Eylül University Child Allergy and Immunology clinic were identified. Patients’ demographic characteristics, clinical and laboratory findings (total IgE, Aspergillus fumigatus specific IgE, eosinophil count, SPT, and spirometry values), treatment protocols were evaluated retrospectively. Predisposing disease in patients was determined. From these diseases; diagnosis of asthma was made with the guidelines of Global Initiative for Asthma^1,^ Global Initiative for Asthma (2020). Global Strategy for Asthma Management and Prevention [online].Website https://ginasthma.org/gina-reports/gina-2020-full-report_-final-_wms/ [accessed 03 April 2020]. and the diagnosis of cystic fibrosis (CF) was made with the American Council of Cystic Fibrosis 1998 consensus report [8]. We used the diagnostic criteria of Agarwal R et al. for the diagnosis of ABPA and the diagnostic criteria of Denning DW et al. for the diagnosis of SAFS [6,7]. The diagnostic criteria of ABPA were; the presence of predisposing disease (Asthma or cystic fibrosis, COPD, post-TB fibrocavitary disease) and with two obligatory criteria that’s are AF specific IgE > 0.35 kU/L or positive AF skin prick test (SPT) and total serum IgE > 1000 IU/mL; and with at least 2 of 3 the other criteria, the presence of radiological findings compatible with ABPA or serum AF IgG > 27 mg/L or total eosinophil count >500 /µL We evaluated the situations that might be a risk factor for the development of ABPA in patients [6]. If the SPT ≥ 3 mm and allergen specific IgE ≥ 0.35 kU/L, the presence of atopy was shown. Extracts of 16 common allergens were used for the prick test (Allergopharma, Merck KGaA, Darmstadt/Germany). Allergen extracts included household mites (Dermatophagoides farinae, Dermatophagoides pteronysinus), grass pollen mixtures (Artemisia vulgaris, Grasses, Parietaria officinalis), tree pollen mixtures (Alnus glutinosa, Betula alba, Fagus silvatica, Populus alba, Olea europaea), mold-mix (Alternaria alternata, Aspergillus fumigatus, Cladosporium herbarum), and animal dander (Blatella germanica, Canis familiaris, Felis domesticus). We classified our patients with the diagnostic scoring and staging criteria suggested by Agarwal et al. ABPA total disease score was obtained by summing the radiological and immunological scores (Table 1) [6].

**Table 1:** Recommended scoring system for the diagnosis of allergic bronchopulmonary aspergillosis (ABPA)

Immunological scoring	Value	Score
A. fumigatus-specific IgE(kUA/L)	<0.350.35–1.9>1.9	–7+1+3
Total IgE(IU/mL)	<417417–10001000–2300>2300	-3+1+2+3
Absolute eosinophil count (cells/µL)	<500500–1000>1000	+0+3+4
A. fumigatus-specific IgG(mgA/L)	<27>27	+0+4
Radiological scoring (Thorax high resolution computed tomography)	Normal	+0
	≥2 features of fibrosis	+2
	Bronchiectasis involving <3 lobes	+3
	Bronchiectasis involving ≥3 lobes	+4
	Extensive mucoid impaction	+4
	Hyperattenuating mucus	+5
Total score	ABPA risk	
Radiological score 0, Total 8	ABPA-S (serological ABPA)	
Radiological score 0, Total ≥9	ABPA-CPF (ABPA with chronic pleuropulmonary fibrosis)	
Radiological score 2, Total ≥9	ABPA-B (ABPA with bronchiectasis)	
Radiological score 3 or 4, Total ≥9	ABPA-HAM (ABPA with high attenuated mucus)	
Radiological score 5, Total ≥9		

The staging of ABPA is as follows:

Stage 0 (Asymptomatic); Asymptomatic cases who have not previously been diagnosed with ABPA, meeting ABPA diagnostic criteria.

Stage 1 (Acute), ABPA compliant with uncontrolled asthma/symptoms.

Stage 2 (Response), 25% or more reduction in total IgE at week 8 of treatment in addition to clinical and/or radiological improvement.

Stage 3 (Exacerbation), increases clinically and radiologically with worsening 50% or more compared to the total IgE level determined in the treatment response/remission. Clinical worsening was accepted as an increase in cough, sputum and respiratory distress.

Stage 4 (Remission), continuous clinical-radiological improvement and total IgE level below baseline (or less than 50% increase) 6 months after treatment.

Stage 5.

Stage 5a (Treatment-dependent), ≥2 exacerbation within 6 months after stopping treatment or worsening of the clinical and/or radiological condition, along with immunological worsening (rise in IgE levels) on tapering oral steroids/azoles.

Stage 5b (Corticosteroid-dependent asthma), Systemic glucocorticoids required for control of asthma while the ABPA activity is controlled (as indicated by IgE levels and thoracic imaging).

Stage 6 (Advance), Extensive bronchiectasis due to ABPA on chest imaging and complications (cor pulmonale and/or chronic type II respiratory failure).

We evaluated thorax high resolution computed tomography (HRCT) findings for both diagnosis and radiological staging. We recorded the values FVC (forced vital capacity), FEV1 (forced expiratory volume in 1 s) and FEV1/FVC with spirometry. During the follow-up of the patients’ clinical, laboratory, imaging and spirometric findings, treatment regimens, treatment responses, and side effects were recorded. 

Categorical data were expressed as numbers, percentages (%), continuous data as mean ± standard deviation, or median (minimum-maximum). Statistical analyses of the study were performed using IBM SPSS Statistics for Windows, v. 25. This study was approved by Dokuz Eylül University Non-Interventional Research Ethics Committee (2020/21-28, 14.09.2020).

## 3. Results

The mean age of the 6 patients was 14.66 ± 1.52 years. Three of these patients were diagnosed with asthma, and the other three were diagnosed with cystic fibrosis. The average age of diagnosis in cystic fibrosis patients was 4.33 ± 1.52 months, while in asthma patients it was 10.66 ± 5.50 years. The median follow-up month of the patients after ABPA diagnosis was 7 (min-max: 4–12) months. The demographic findings of the patients are summarized in Table 2. 

**Table 2:** Demographic findings of the patients.

	Case 1	Case 2	Case 3	Case 4	Case 5	Case 6
Age (year)	13	16	15	12	17	13
SexFemale/Male	F	F	M	F	M	F
Predisposing condition	Cystic fibrosis	Cystic fibrosis	Cystic fibrosis	Allergic asthma-SAFS	Allergic asthma	Allergic asthma -SAFS
Predisposing condition diagnosis age(month/year)	3m	6m	4m	7y	17y	8y
Genetic disorder	Homozygous p.Phe508 del	Compound heterozygous p.G542X, p.N1303K	Compound heterozygous p.Phe508del p.N130K	-	Heterozygousp.I556V	-
Bacterial colonization	-	Pseudomonas aeruginosa	Staphylococcus aureus	-	-	-
Presence of atopy in the patient(nonfungal)	-	+	+	+	+	+
Spirometry (initial)						
FEV₁	95	-	44	80	102	67
FVC	104	-	67	78	108	85
FEV₁/FVC	91		65	102	91	78
The presence of atopy in the family	+	-	-	+	-	+
Relationship between parents	+	-	+	-	-	-

At the time of ABPA diagnosis; the median total IgE level was 1033 IU/mL (1004–6129), the median absolute eosinophil count (AEC) was 566/µL (200–800). Only in our third case, AEC was lower than 500/µL, however, our patient had high values in the past. Median AF specific IgE mean of the patients was 10.64 kU/L (2.59–49.70). IgG specific to A. fumigatus could not be observed in any of our patients. The total score could be calculated in all of our patients except for the 4th patient since it was ≥ 9 independent of the AF specific IgG parameter. The total scores of our other patients were between 10 and 12, and were consistent with the diagnosis of ABPA-B. Diagnostic scores and laboratory findings calculated when patients were diagnosed with ABPA are summarized in Table 3.

**Table 3:** Diagnostic scores and laboratory findings of patients at admission.

	Case 1	Case 2	Case 3	Case 4	Case 5	Case 6
Immunological score	8311ABPA-B*	8412ABPA-B*	6410ABPA-B*	-0--	8412ABPA-B*	8412ABPA-B*
Radiological score						
Total score						
Scoring result						
Total IgE (IU/mL)	1004	1944	6129	1036	1004	1031
AEC† (/uL)	600	500	200	800	600	700
AF Specific IgE‡ (kU/L)	19.20	49.70	7.09	1.99	14.20	2.59

*: Allergic bronchopulmonary aspergillosis with bronchiectasis, †: Absolute eosinophil count, ‡: Aspergillus fumigatus

The skin prick tests of our patients are summarized in Table 4.

**Table 4:** Skin prick yest (SPT) results of patients

SPT (mm)	Case 1	Case 2	Case 3	Case 4	Case 5	Case 6
Positive control	3	4	3	3	3	4
Weed/Grain pollen	-	7	7	8	4	4
Olive pollen	-	8	-	8	-	-
Alder pollen	-	-	-	-	4	-
Cat dander	-	-	-	5	-	-
A. alternata*	-	-	-	8	4	7
C.herbarum†	-	-	-	4	-	3
A. fumigatus‡	5	7	4	4	5	4

*: Alternaria alternata, ‡: Aspergillus fumigatus, †: Cladosporium herbarum

**Figure 1:**
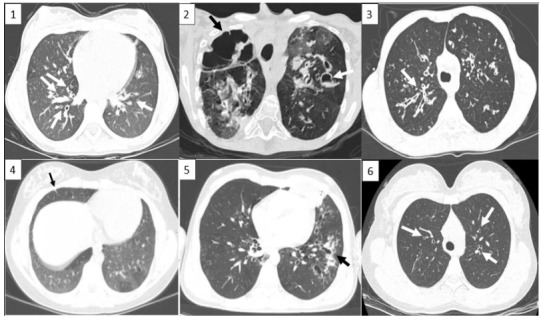
High-resolution computed tomography images of the patients. 1a. Peribonchial thickening, 1b. Cystic areas, 1c. Budding tree sign, 1d. Nonspecific nodule, 1e. Pulmonary infiltration, 1f. Central bronchiectasis.

Except for one case (case 4), bronchiectasis was detected in HRCT of 5 other cases. Mosaic perfusion areas, peribronchial thickening, cystic lesions, budding tree sign, nonspecific nodule, pulmonary infiltration were other remarkable radiological findings. HRCT findings of our patients are shown in Figure 1.

In spirometry, the rate of FVC, FEV1 and FEV1/FVC was found to be low in our 3rd and 6th patients while it was over 80% in our other patients (Table 2). We detected an increase of approximately 20% in FEV1 levels after the first month of ABPA treatments in our 3rd and 6th cases, and this improvement continued throughout their clinical follow-ups. In our second case, spirometric evaluation could not be performed due to the poor general condition.

We preferred a high dose of ICS and oral itraconazole treatment in patients with asthma with ABPA. Treatment regimens vary in our cystic fibrosis patients. Treatment protocols and doses of patients are shown in Table 5.

**Table 5:** Treatment protocols of patients.

	Treatment protocol
Case 1	High-dose inhaled fluticasone propionate* and oral itraconazole‡
Case 2	Oral methyl prednisolone† and oral itraconazole‡
Case 3	Oral methyl prednisolone† and posaconazole§ → omalizumab||
Case 4	High-dose inhaled fluticasone propionate/salmeterol¶ and montelukast and oral itraconazole‡
Case 5	High-dose inhaled fluticasone propionate* and oral itraconazole‡
Case 6	High-dose inhaled fluticasone propionate/salmeterol¶ and montelukast and oral itraconazole‡

*: Fluticasone propionate 1 mg/day.‡: Oral itraconazole 5 mg/kg (max: 400 mg/day)- about 16 weeks/up to 1 month after steroid cut. †: Oral methyl prednisolone. Beginning with 2 mg/kg/day, reducing the dose every 2 weeks, going over everyday and cutting after 3 months.§: Posaconazole. First day 2 x 300 mg, then 1 x 300 mg, 6–12 weeks||:

Our third case used oral methylprednisolone for about 5 months with ABPA diagnosis approximately 2 years ago, 6 months after the cessation of itraconazole treatment, and the same treatment protocol used for the second time for 9 months with the diagnosis of relapse ABPA. Due to the development of an ABPA attack for the third time 3 months after the treatment, we started the treatment of oral methylprednisolone and posaconazole. However, although we saw the desired total IgE decrease in the first month of treatment, we planned a treatment change due to the development of steroid-related enterocolitis. For this reason, we started the subcutaneous omalizumab treatment. Spirometric values increased to >80% after the first month of treatment, and significant improvement in clinical findings was observed from the 2nd week. 

**Figure 2:**
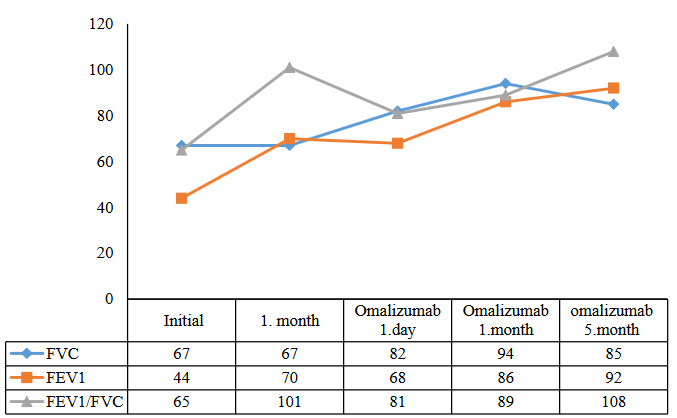
Spirometric changes in the follow-up of our third case.

Total IgE levels of our cases were recorded at the time of diagnosis and during their follow-up. We learned that our fourth case voluntarily stopped treatment in the 2nd month of treatment and later had asthma attacks. We followed up again by ensuring patient compliance. Since our second case died, it could not be followed up. Figure 2 shows the changes in total IgE levels during the follow-up of the cases.

## 4. Discussion

ABPA is defined by various clinical and immunological responses to Aspergillus fumigatus antigens [9]. Long-term azithromycin therapy; bacterial bronchial colonization; use of high-dose inhaled corticosteroids; genetic factors such as CFTR gene mutations and the presence of atopy and eosinophilia have been reported to increase the risk of ABPA [10]. Similar risk factors were present in our patients, as seen in Table 2. Our fourth and sixth cases were diagnosed with SAFS. Two of our 3 patients who developed ABPA with CF had eosinophilia. We think that the presence of atopy and eosinophilia in cystic fibrosis patients may be risk factors for ABPA and care should be taken in terms of the development of ABPA in these patients. Three of our patients who developed ABPA with asthma had fungal sensitivity and two were being followed up for severe asthma. Because of the possibility that patients with similar characteristics may be at increased risk of developing ABPA and clinical progression may be faster than in other asthma groups, close monitoring may be needed in the follow-up of these patients.

The criteria defined in adults are used for the diagnosis of ABPA in children. However, the cut-off value of total IgE for ABPA is not well defined in children. In the study conducted in children between the ages of 5**–**15, total IgE 1204 IU/L and AF specific IgE 0.49 KUA/L were recommended for the diagnosis of ABPA [11]. In another study with an average age of 35.9, it was reported that the sensitivity of A. fumigatus specific IgE >1.91 kUA/L, total IgE >2347 IU/mL and total eosinophil count >507 cells/µL cut-off values were 70% and specificity was 100% in asthma patients [12]. Our patients’ total IgE levels were in the range of 1004**–**6129 IU/L and Aspergillus specific IgE levels were in the range of 1.99**–**49.7 kU/L. We found the highest total IgE and aspergillus specific IgE levels in our patients with CF. However, in order not to delay the diagnosis of ABPA or to prevent unnecessary long-term drug use, we think that extensive studies are needed to clarify the cut-off values according to the young age groups and predisposing disease.

For the diagnosis of ABPA disease, a scoring system including radiological and immunological scores was proposed [6]. The scoring result of our 5 patients was consistent with ABPA-B diagnosis. Due to the lack of sharp cut-off values, this scoring system can be applied more easily in paediatric patients compared to current diagnostic criteria, but care must be taken in the differential diagnosis.

The most obvious radiological findings in ABPA are bronchiectasis and pulmonary infiltration. Glove finger appearance caused by secretion-filled bronchi, train rail appearance, lobar collapse and local consolidation, are other radiological findings. HRCT has been the preferred radiological imaging method in ABPA because of its high sensitivity in the diagnosis of bronchiectasis and showing other abnormalities well [6]. We detected bronchiectasis in 5 of HRCT images taken at the time of diagnosis. Paediatric patients are afraid of tomography shots because of their potential risks. As an alternative to this diagnostic imaging method, we thought it might be useful to evaluate the position of ultrasonic imaging methods in diagnosis and follow-up in terms of findings that cannot be detected by AC radiography.

**Figure 3:**
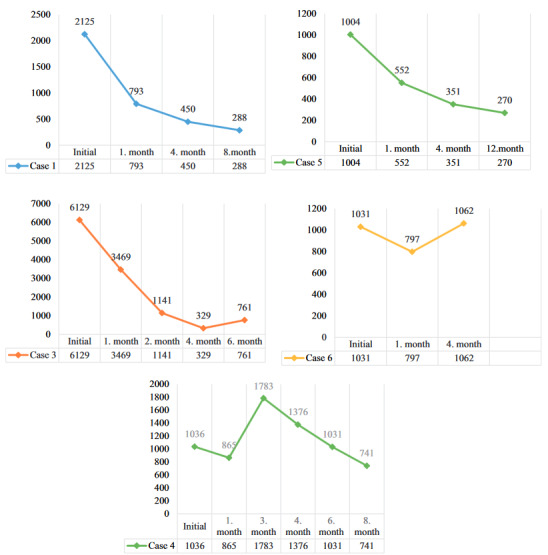
Changes in total IgE levels of cases.

Spirometry helps to show obstruction in airflow, however, normal spirometry does not exclude asthma or ABPA diagnosis [13]. However, it is a useful tool for monitoring lung functions during follow-up [14]. Except for our third case, where spirometric measurements of our patients were not very low despite their clinical picture. The FEV1 and FEV1/FVC values of our third case were significantly increased with one month of oral methylprednisolone and posaconazole treatment, but no change was detected in FVC. At the 24th hour of the first dose of omalizumab treatment, FVC improved markedly and it was observed that all parameters exceeded 80% in the 1st month (Figure 3).

Agarwal et al. proposed a clinical staging in patients with ABPA. At the time of diagnosis, only our third patient was compatible with Stage 5a (treatment-dependent) and the others were in Stage 1 (Acute). Identification and close follow-up of patients in the asymptomatic stage will be important to prevent the progression of the disease [6].

It has also been reported in studies that a cut-off value of 1000 IU/mL can help differentiate SAFS from ABPA-S. It was emphasized that these patients can be labeled as ‘ABPA- at risk’ and close monitoring and follow-up should be performed [15]. In our 4 and 6 cases in the SAFS clinic, we found that the symptom control was insufficient despite the 5th step treatment. We concluded that the addition of itraconazole therapy would be beneficial in preventing chronic lung findings in patients ABPA with asthma who have not received the desired clinical response despite the increase of step therapy. Extensive research is still needed to investigate the relationship between SAFS and ABPA.

There are treatment recommendations according to stages in ABPA. Oral glucocorticosteroids recommended in Stage 1 cases may be reduced and discontinued in Stage 2 while the patient is in remission. Itraconazole is recommended to be added to treatment in patients with relapse (stage 3) or in patients with corticosteroid-dependent asthma (stage 4). It is not clear whether any treatment is effective in patients who develop end-stage pulmonary fibrosis [16,17]. However, systemic corticosteroids and antifungals have often failed due to inadequate symptom control and undesirable side effects; consequently, alternative treatments have been investigated [18]. Long-term oral steroid therapy is particularly problematic for CF patients who are already prone to diabetes, osteopenia and growth retardation [19]. Omalizumab is used in patients with severe allergic asthma, with a steroid-protective effect. Based on these results, it has been suggested that omalizumab may be beneficial in patients with ABPA [20]. While high dose ICS and oral itraconazole treatment was preferred in our patients with asthma with ABPA, treatment regimens were different in patients with CF. We preferred the combination of OCS and itraconazole in our patient with a poor clinical condition and the combination of ICS and itraconazole in our patient with aeroallergen sensitivity. Oral corticosteroids (OCS) and posaconazole treatment was discontinued in our patient who came with relapse and then omalizumab treatment was started. The place of ICS in ABPA treatment was evaluated in a review. While ICS treatment was effectively detected in several case reports and small case series, it was reported to be of no benefit in a double-blind multicenter placebo-controlled study [6]. In addition to being an antifungal, itraconazole increases the systemic bioavailability of inhaled corticosteroids. Therefore, it has been assumed that this effect of itraconazole causes a clinical response but increases the potential side effects of the steroid [21,22]. We preferred ICS and itraconazole treatment in four of our patients and we did not encounter any side effects. In our patient using OCS, enterocolitis developed and we thought that the patient who had growth retardation due to CF may have additional problems due to steroid use. We have experienced in our patient that ICS, itraconazole treatment is beneficial in patients with aeroallergen sensitivity and caught at an early stage. Therefore, we think it would be beneficial to re-evaluate this combination in large patient populations.

The effects of omalizumab treatment on spirometry have led to contradictory results in studies. In some studies, FEV1 (forced expiratory volume in 1 s) and FVC (forced vital capacity) values did not improve, while some case reports showed improvement [23]. In our third case, we observed a significant improvement in FEV1 and FVC when we compared the onset and 24th hour of Omalizumab treatment. And this improvement continues with subsequent doses of omalizumab. 

During ABPA treatment, control of chest radiography and serum total IgE levels are recommended every 6**–**8 weeks [6]. We evaluated the clinical findings, total IgE levels and chest radiographs after 1 month of treatment. Our fourth patient had a 16% decline in the total IgE level, while the others were 25% and above. Treatment change was not considered in our fourth patient due to the good clinical condition and radiological findings. However, we associated more than 50% increase in 3rd-month control with the patient’s treatment mismatch. With the reorganization of the treatment, we detected a 23% decrease in the 1st month. While our third case saw a dramatic decrease in the total IgE level up to the 4th month, we observed that the 6th month increased more than twice the last level. We did not plan a treatment change considering that total IgE level was affected by omalizumab treatment and the presence of atopy due to no clinical or radiological deterioration. Even though there was a 33% increase in the total IgE level in the 4th month of the treatment of our sixth case, we thought that this situation was related to seasonal weed pollen sensitivity due to the absence of complaints and clinical findings. Especially in patients with allergen sensitivity and using Omalizumab therapy, it is necessary to decide that the total IgE level is misleading, considering the other findings of the patient. We think that it would be beneficial to bring a different parameter to be used in the follow-up of these patients.

In conclusion; even though immunopathogenesis, genetic, and environmental risk factors for ABPA have been investigated, they have not yet been revealed clearly. ABPA diagnostic criteria and treatment regimens have not yet been customized for paediatric patients. For this reason, considering the underlying predisposing disease in paediatric patients, cutt-off values, diagnostic criteria and scoring systems for total IgE, AF-specific IgE, AEC should be determined. Thus, by developing treatment regimens suitable for clinical stages, patients can be protected from drug side effects.

## Informed consent

The study was carried out in accordance with the principles of the Helsinki Declaration. As a routine procedure, written informed consent was obtained from each patient for all procedures and publication. This study was approved by Dokuz Eylül University Non-Interventional Research Ethics Committee (2020/21-28, 14.09.2020).
